# Club Cell Loss as a Feature of Bronchiolization in ILD

**DOI:** 10.3389/fimmu.2021.630096

**Published:** 2021-02-26

**Authors:** Paul Reynaud, Engi Ahmed, Isabelle Serre, Lucie Knabe, Sébastien Bommart, Carey Suehs, Isabelle Vachier, Jean Philippe Berthet, Micaela Romagnoli, Charlotte Vernisse, Jean Pierre Mallet, Anne Sophie Gamez, Arnaud Bourdin

**Affiliations:** ^1^Department of Respiratory Diseases, Univ Montpellier, CHU Montpellier, Montpellier, France; ^2^PhyMedExp, Univ Montpellier, CNRS, INSERM, CHU Montpellier, Montpellier, France; ^3^Department of Pathology, Univ Montpellier, CHU Montpellier, Montpellier, France; ^4^Department of Radiology, Univ Montpellier, CHU Montpellier, Montpellier, France; ^5^Pulmonology Unit, Ospedale Ca'Foncello, AULSS2 Marca Trevigiana, Trevise, Italy; ^6^Department of Cardiac, Thoracic and Vascular Surgery, Univ Nice, CHU Nice, Nice, France

**Keywords:** club (clara) cell, idiopathic pulmonary fibrosis, metaplasia, SCGB1A1, bronchiolization, interstitial lung disease

## Abstract

**Background:** Distal airway metaplasia may precede honeycombing in progressive fibrosing interstitial lung disease (ILD). The SCGB1A1^+^ bronchiolar-specific club cell may play a role in this aberrant regenerative process.

**Objective:** To assess the presence of club cells in the small airways of patients suffering from ILD.

**Methods:** Small airways (internal diameter <2 mm) in lung samples [surgical lung biopsy (SLB) and/or transbronchial lung cryobiopsy (TBLC)] from 14 patients suffering from ILD and 10 controls were morphologically assessed and stained for SCGB1A1. SCGB1A1 was weighted by epithelial height as a marker of airway generation (SCGB1A1/EH). Correlations between clinical, functional, and high-resolution CT (HRCT) prognostic factors and histomorphometry were assessed.

**Results:** Small airways from samples with ILD patterns were significantly less dense in terms of SCGB1A1^+^ cells [0.064 (0.020–0.172)] as compared to controls' sample's small airways [0.393 (0.082–0.698), *p* < 0.0001]. Usual interstitial pneumonia (UIP) patterns most frequently contained small airways with limited or absent SCGB1A1 expression (SCGB1A1/EH <0.025): UIP (18/33; 55%) as compared with non-UIP patterns (4/31; 13%) or controls (0/29; 0%): *p* < 0.0001. In addition, correlations with HRCT indicated a significant negative relationship between SCGB1A1 and bronchiectasis as a feature of bronchiolization (Rho −0.63, *p* < 0.001) and a positive relationship with both forced vital capacity (FVC) and Hounsfield unit (HU)-distribution pattern in kurtosis (Rho 0.38 and 0.50, respectively, both *p* < 0.001) as markers of fibrotic changes.

**Conclusion:** Compared with controls, the small airways of patients with ILD more often lack SCGB1A1, especially so in UIP. Low densities of SCGB1A1-marked cells correlate with bronchiectasis and fibrotic changes. Further research investigating SCGB1A1 staining as a pathological feature of the bronchiolization process is merited.

## Introduction

Interstitial lung disease (ILD) are devastating affections nearly constantly leading to respiratory failure and death. The early phases of the disease are hallmarked by progressive fibrosis in the peripheral zones of the lung, which is associated with lung function decline (1). High-resolution CT (HRCT) has become the gold standard for diagnosing such fibrotic changes and characterizing their progression (2). Notably, the presence of traction bronchiectasis (the enlargement of small airways by traction) is now considered a red flag for subpleural fibrotic changes. Interestingly, histomorphological assessments of these fibrotic areas consistently report bronchiolar metaplasia of unclear origin (3). The latter “bronchiolization” may represent aberrant regeneration of the lung in a profibrotic environment, where distal airway precursors never reach their potential as functional alveoli (4). The result is the presence of proximal features in the distal compartment currently detectable only *via* histopathology (5).

Club cells are thought to be highly expressed at the small airway level (6), where they are supposed to play a protective role against noxious inhaled particles. Among the latter, cigarette smoke and occupational dust have also been implicated in the pathogenesis of idiopathic pulmonary fibrosis (IPF) (7, 8). Interestingly, club cells were also attributed an accessory stem cell role at the small airway level, but this role has mostly been evidenced in animal models at the bronchoalveolar duct junction, where they are preferentially located (9). Due to recent advances in single-cell biology, it is now clear that these cells are heterogeneous and not all are playing the same role. Currently, they are considered as differentiation of basal cells, and the transition from basal to club cells has been ontogenically evidenced. Additionally, club cells are situated at the crossroads of the epithelial phenotype. Their natural trend is to progressively acquire features of goblet cells, but some can also move toward ciliogenesis. Whether or not appropriate transition pathways are failing during the bronchiolisation process remain unclear.

New opportunities for lung tissue sampling are provided by the arrival of transbronchial lung cryobiopsies (TBLC). Beyond clinical and technical considerations, it seems that TBLC, as compared to traditional surgical lung biopsies (SLB), samples airway-centered, more proximal tissue, and this is likely to change not only the pathological patterns and eventually the final pathological diagnosis but also the representation of the different compartments. In particular, airways contained in TBLC samples are more likely to represent earlier generations than those taken in the more peripheral SLB samples (10). Intuitively, the small airways present in TBLC samples might then be less frequently affected by bronchiolization. Given the relationship between this process and the fibrotic changes affecting the lung in progressing ILD, these pathological changes may have some diagnostic and/or prognostic value identifiable by clinical correlations in a cross-sectional study.

The objective of this study was to assess the presence of club cells in the small airways of patients suffering from ILD by combining pathology, physiology, and HRCT data.

We hypothesized that “protective” club cells characterized by their specific protein SCGB1A1 might be deficient in fibrotic diseases and particularly at the site where bronchiolar metaplasia occurs.

## Materials and Methods

CryoPID [NCT02763540] was a prospective trial evaluating the diagnostic concordance between TBLC and SLB, which were performed sequentially in the same patients between January 2016 and March 2018 at the Montpellier University Hospital (11). The study included patients with ILD requiring an SLB as per a multidisciplinary approach decision, given an undefined HRCT pattern. For the current histology assessment, 14 CryoPID patients were compared with 10 control subjects randomly selected from the bio-bank of the Montpellier University Hospital. Pathological patterns reported from SLB and TBLC together by a pathologist involved in the present study were used. Controls had normal lung function, were free of chronic respiratory disease, and matched, as well as a possibility for smoking, in gender and age with the ILD population. They had undergone a lobar resection for a colic metastasis in the last 3 years. Lung parenchyma at a large distance from the metastasis was analyzed.

Briefly, lung samples were fixed in 4% formalin at pH 7.4 and embedded in paraffin. Three-millimeter-thick sections were stained by H&E. Lung samples were anonymized and blindly assessed by an expert pathologist (TVC). Serial sections were used for SCGB1A1 immunostaining (dilution 1/10,000; Biovendor, Czech Republic). For some slides, a Bleu Alcian staining could also be performed.

**Figure 1:**
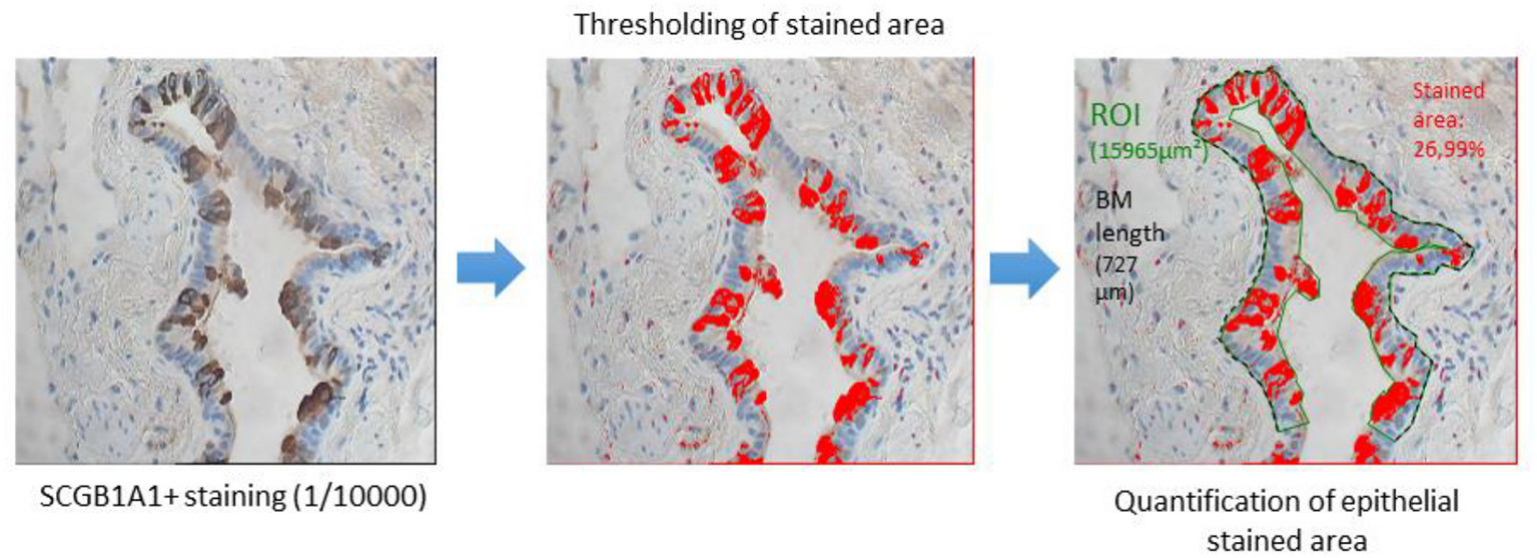
After SCGB1A1 staining, small airway epithelia were isolated *via* thresholding, perimeters, thickness/height, and by the area quantified.

Small airways (identified by an internal diameter of 2 mm or less) were assessed in all samples. Morphometry was assessed using the Cell P software (Olympus, Tokyo, Japan). Bronchiolization was referred to small airways with evidence of proximal features, including the presence of ciliated and/or goblet cells, as per the previously published standards (12, 13). Epithelial area, epithelial height (EH), and the percentage of SCGB1A1^+^ immune-stained area were determined for each small airway. The SCGB1A1^+^/EH ratio was computed to adjust for variations known to affect club cell density through airway generation (Figure 1). The small airway density for each sample was based on a semi-quantitative scale ranging from 0 (absence of small airways) to 5 (highest small airway density). The absence or near-absence of club cells was defined by a SCGB1A1/EH ratio < 0.025 by taking into account the background noise.

Prognostic factors were recorded and included clinical (age, gender, smoking history), biological/laboratory [arterial oxygen pressure (PaO_2_), bronchoalveolar lavage cellularity (BAL)], and functional [forced vital capacity (FVC), diffusing capacity of the lungs for carbon monoxide (DLCO)] criteria.

A visual tomodensitometric analysis scored fibrotic changes (honeycombing and reticulations), intensity, and the spread of ground-glass opacity (GGO) (14), as well as the extent of bronchiectasis [ranking the severity of bronchiectasis by a lobe on a scale ranging from 0 to 3 (15) by acquiring consensus among two experienced thoracic senior radiologists (SB and GD)].

Automatic quantification *via* the Thoracic VCAR software (GE HealthCare, USA) was also performed, classifying pixels from the whole lung (WL) volume into four groups as proposed by Shin et al. (16) (< −950 HU: “emphysema,” −950 to −700 HU “functional parenchyma,” −700 to −500 HU “interstitial disease,” >−500 HU “extra parenchymal”). Three prognostic variables were then derived: total lung capacity (TLC), percentage of normal lung (%NL), and mean lung attenuation (MLA) (17). Pulmonary attenuation distribution histograms were obtained for assessing skewness and kurtosis, previously reported as having the prognostic value (18).

### Statistical Analysis

Parametric and non-parametric tests were used to describe and compare normally and non-normally distributed data as appropriate. Spearman's correlation coefficients were computed for testing variable relationships.

## Results

### Population Description

Fourteen patients with ILD and 10 controls were studied (Table 1). As expected, patients with ILD had worse pulmonary function tests and higher HRCT semi-quantitative scores. Of note, as these patients were deemed eligible for SLB, most had a mild functional impairment, suggesting that many were at an early stage of the disease. As previously reported (11), discrepancies in the pathological diagnosis between SLB and TBLC existed, potentially leading to different final diagnoses during a second multidisciplinary assessment (Table 2). For subsequent classification as usual interstitial pneumonia (UIP) and non-UIP, only samples with a clear and confirmed (high level of confidence) UIP pattern were retained.

**Table 1:** Population characteristics.

	**ILD [Median (IQR25–75)]**	**Controls [Median (IQR25–75)]**	***p*-value**
*N*	14	10	
Age (years)	63 [43.5–65]	64 [57.5–67.5]	0.21
Gender (W/M)	10 vs. 04	3 vs. 7	0.0987
Weight (kg)	65.5 [60.65–81.75]	65 [62.25–83.75]	0.49
Height (cm)	160 [158.5–170]	166.5 [160–170]	0.44
Smoking history (p.y)	2.5 [0–28.75]	19 [1.25–30]	0.77
Smoking status (current-former-never)	1/5/8	1/5/4	
Positive autoantibodies	0	0	
PaO_2_ (mmHg)	84.2 [75.75–88.28]	88.65 [81.29–93.5]	0.15
FVC (l)	2.3 [1.74–3.31]	3.43 [2.75–4.07]	0.027^*^
FVC (% pred values)	84.5 [68.5–2.77]	100 [92.25–107.5]	0.004^*^
FEV1 (l)	2.1 [1.59–2.77]	2.39 [2.02–3.39]	0.077
FEV1 (% pred values)	84 [72.75–90]	95.5 [86–110.8]	0.010^*^
TLC (l)	3.69 [3.55–4.49]	6.23 [6.16–6.59]	0.001^*^
TLC (% pred values)	77.5 [66.25–84.75]	103 [101–106]	0.00005^*^
RV (l)	1.57 [1.31–1.79]	2.64 [2.58–3.12]	0.0002^*^
RV (% pred values)	89 [65–102]	116 [100–121]	0.0049^*^
TLCO (% pred values)	56 [40–60]	76 [70–80]	0.0097^*^
KCO	79 [73–96]	85 [78–103]	0.58
Kurtosis	6.33 [4.32–7.48]	14.77 [13.78–17.16]	0.0017^*^
Skewness	2.29 [2.02–2.53]	3.59 [3.39–3.85]	0.00014^*^
Whole lung (WL)	3.44 [2.79–4.12]	5.61 [3.54–6.17]	0.1095
% Normal lung (NL)	79.98 [71.14–82.04]	88.72 [88.2–90.25]	0.00045
Mean lung attenuation (Hounsfield)	−764 [−796 to −720]	−838 [−859 to −819]	0.0012^*^
Fibrotic score	15 [10–22.5]	0 [0–0]	0.00006^*^
Ground glass opacity	5 [5–5]	0 [0–0]	0.059
Bronchiectasis score	6 [5.25–8]	0 [0–0]	0.00003^*^

%NL, percentage of normal lung; BMI, body mass index; CT, computed tomography; DLCO, diffusing capacity of the lungs for carbon monoxide; FEV1, forced expiratory volume in 1 s; FVC, forced vital capacity; ILD, interstial lung disease; IQR, interquartile range; KCO, transfer coefficient of the lung for carbon monoxide; MLA, mean lung attenuation; N, number; PaO_2_, arterial oxygen pressure; RV, residual volume; SD, standard deviation; TLC, total lung capacity.

* *p < 0.05*.

**Table 2:** Blinded histology and multidisciplinary diagnostic results for each patient.

**Patient**	**Histology diagnosis based on TBLC**	**Histology diagnosis based on SLB**	**Diagnosis based on MDA 1 (before histology)**	**Diagnosis based on MDA 2 (after histology)**
1	UIP	UIP	Possible UIP	CHP
2	Non-diagnostic	PLCH	DIP	PLCH
3	UIP	CHP	NSIP	IPF
4	UIP	NSIP	No classification	NSIP
5	RB-ILD	RB-ILD	RB-ILD	RB-ILD
6	Non-diagnostic	UIP	NSIP	IPF
7	PLCH	UIP	Possible UIP	IPF
8	NSIP	NSIP	Fibrotic NSIP	NOS
9	UIP	UIP	NSIP	IPF
10	Non-diagnostic	ALI	NSIP	NOS
11	NSIP	LP	Sarcoidosis	CVID
12	UIP	NSIP	NSIP	NSIP
13	Non-diagnostic	UIP	CHP	IPF
14	UIP	UIP	Possible UIP	IPF

*CHP, chronic hypersensitivity pneumonitis; CVID, common variable immune deficiency; DIP, desquamative interstitial pneumonia; HP, hypersensitivity pneumonitis; IPF, idiopathic pulmonary fibrosis; LP, lymphoid process; MDA1, a first multidisciplinary assessment; MDA2, the final multidisciplinary assessment; NOS, not otherwise specified; NSIP, non-specific interstitial pneumonia; OP, organizing pneumonia; PLCH, pulmonary Langerhans cell histiocytosis; RB-ILD, respiratory bronchiolitis-associated interstitial lung disease; SLB, surgical lung biopsy; TBLC, transbronchial lung cryobiopsy; UIP, usual interstitial pneumonia*.

**Figure 2:**
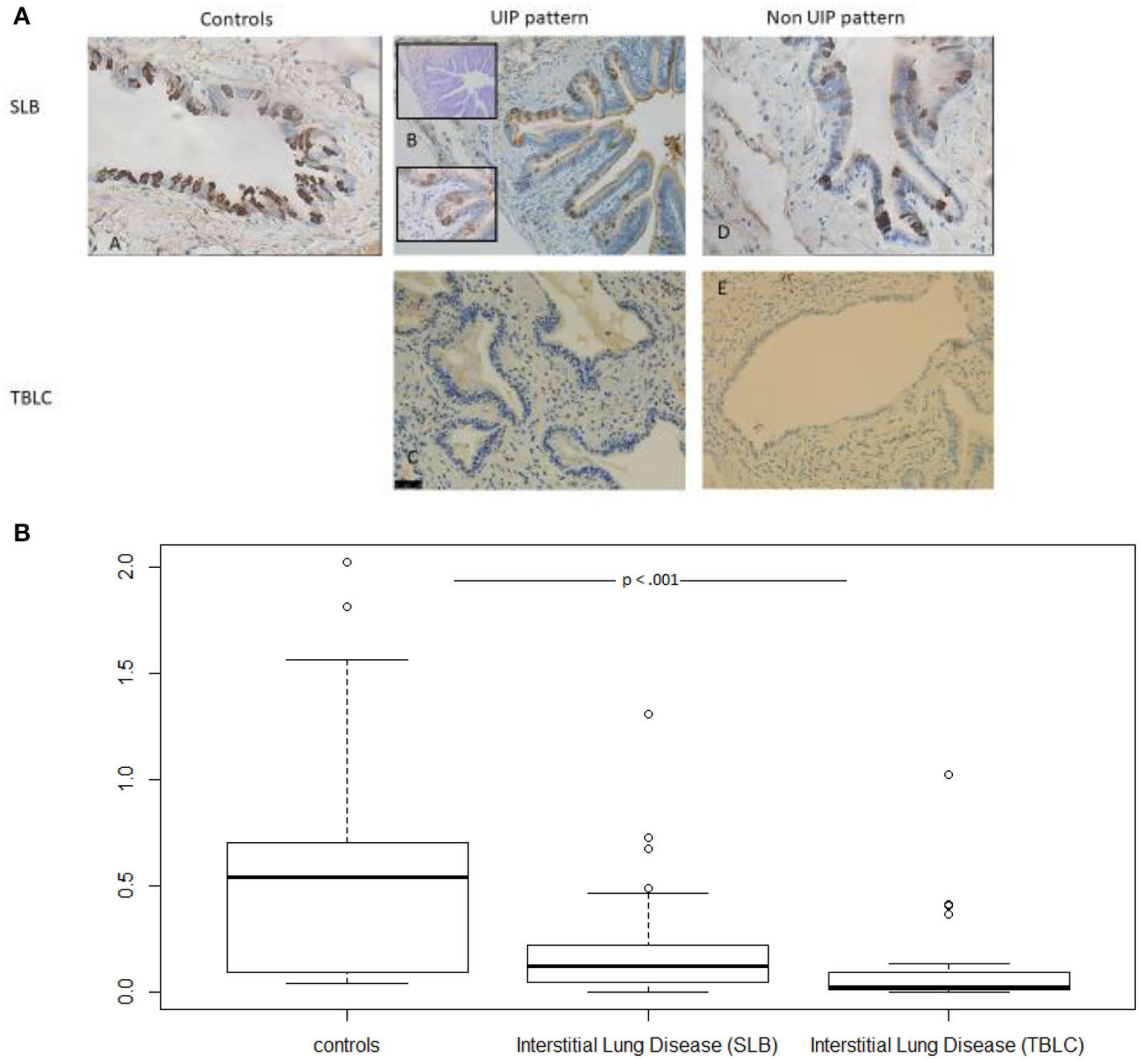
**(A)** Small airways are immune-stained for SCGB1A1 in controls, UIP, and non-UIP patterns. Upper left: (A) SLB from a control (no TBLC were available in this group). Middle row: (B) Very limited staining in some (left lower part) but not all small airways sampled by SLB; Inserts are: Serial cut with a Blue Alcian staining suggestive of the presence of mucin-costaining and a higher magnification suggestive of goblet cell morphology for SCGB1A1 positively stained epithelial cells. Ectasic small airway from a TBLC UIP pattern with bronchiolar metaplasia nearly free of staining despite abundant mucostasis. Right: SCGB1A1 stained small airways obtained by SLB (D) and by TBLC (E) in non-UIP patterns. **(B)** Box plots demonstrating group differences (Control vs. ILD for SLB and TBLC) for SCGB1A1 staining variables. TBLC, transbronchial lung cryobiopsies; SLB, surgical lung biopsy; UIP, usual interstitial pneumonia; ILD, interstitial lung disease.

Among patients with ILD, the mean SLB sample size was 49.5 mm [41.5–60] compared to 7 mm (6–8) (*p* < 0.00001) for TBLC. A significant difference in the semi-quantitative scoring of small airway density between SLB and TBLC sample was found (Table 3). No difference was found between TBLC and SLB regarding club cell representation in the small airways. Fewer airways positively stained for SCGB1A1 in ILD, in particular, for those with a pathological UIP pattern, and in general, this staining was less intense (Figure 2A). This decrease persisted after adjusting on epithelial height as a surrogate marker of the generation of the airway assessed. Hence, SCGB1A1/EH was statistically lower in the small airways of samples from patients with ILD vs. controls (Figure 2B; the Kruskal-Wallis test, *p* = 7.598e-07).

**Table 3:** Samples characteristics.

	**SLB (controls)**	**SLB (ILD)**	**TBLC (ILD)**	***p*-value**
*N*	10	14	14	
Number of small airways assessed	32	35	31	
Small airway density [semi quantitative score (0 = minimal; 5 = maximal)]	2.1 ± 0.1	2.4 ± 0.6	0.4 ± 0.3	0.04
Epithelial area (mean ± SD per bronchiole. μm^2^)	20,818 ± 9,040	26,738 ± 11,733	19,638 ± 11,216	ns
Epithelial height (mean ± SD per bronchiole. μm)	37.8 ± 7.8	39.5 ± 11.9	35.8 ± 11.4	ns
SCGB1A1^+^ stained area	19.7 (4.2–27.7)	3.6 (1.6–7.4)	0.9 (0.3–2.9)	<0.0001
SCGB1A1^+^/EH	0.5 (0.1–0.7)	0.1 (0.0–0.2)	0.0 (0.0–0.1)	0.02
% airway without club cells^§^	0 (0/32)	14 (5/35)	55 (17/31)	0.01

§ * Defined by SCGB1A1+/EH ratio <0.025*.

**Figure 3:**
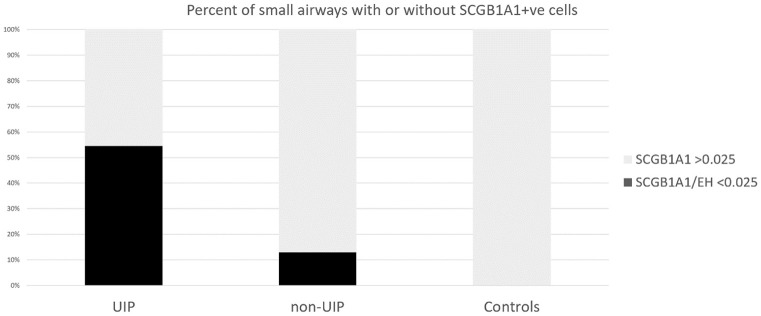
The proportion of samples with SCGB1A1/EH <0.025 was used to define the absence of positive staining [UIP 18/33 vs. non-UIP 4/31 (*p* = 0.0027)].

Moreover, sparsely stained airways (defined as SCGB1A1/EH <0.025) were significantly more represented in the UIP pathological patterns [UIP 18/33 vs. non-UIP 4/31 (*p* = 0.0027)] (Table 2 and Figure 3).

**Figure 4:**
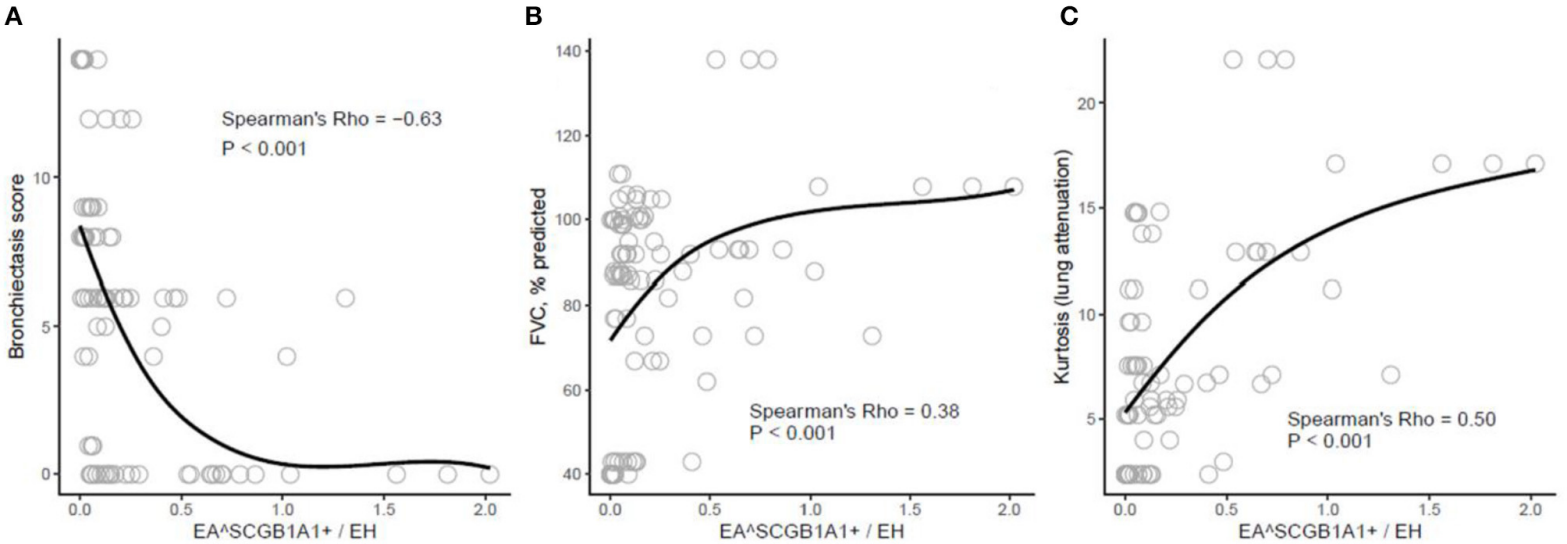
Scatterplots demonstrating the relationships between selected pulmonary function variables [bronchiectasis score **(A)**, % predicted forced vital capacity (FVC, % predicted) **(B)**, and kurtosis of lung attenuation scores **(C)**] and % epithelial area of small airways that stains SCGB1B1^+^ weighted by epithelial height (EA^SCGB1B1+^/EH). Smoothing is performed *via* a local loess estimator (in black). Spearman's correlation statistics (Rho and FDR-corrected *p*-value) are provided.

The tested prognostic correlations are provided in Figure 4. Interestingly, statistically significant non-parametric correlations were found between SCGB1A1/EH and two surrogate markers of lung fibrosis. First, a correlation was found with the kurtosis value of the histogram distribution of lung attenuation assessed at HRCT (Rho = 0.50, *p* < 0.001). The less the lung is involved by fibrosis, the greater the club cell density found in the small airways, as the kurtosis value decreases with the extent of fibrosis in ILD (18). Second, SCGB1A1/EH also positively correlated with FVC (Rho = 0.38, *p* < 0.001), as expected.

The negative correlation found between SCGB1A1/EH and the bronchiectasis scores (Rho = −0.63, *p* < 0.001) is also represented in Figure 4. The latter indicates that fewer club cells were present in the small airways of patients with more intense bronchiectasis.

## Discussion

With the aim of understanding the mechanisms of bronchiolization, we found that club cells are absent or nearly absent from these aberrant small airways in the most subpleural areas of fibrotic lungs. This was particularly true in ILD where a UIP pattern could be identified. Bronchiolized areas seem to fit with small airways saliently featuring proximal differentiation patterns very close to terminal airspaces. As expected, both TBLC and SLB could identify these patterns, even though the density of small airways in TBLC is largely lower. Interestingly, the correlation between these bronchiolization patterns (highlighted by the absence of club cells) and clinical prognostic markers (such as HRCT kurtosis and bronchiectasis), as well as physiology (FVC), was demonstrated.

Most of our results were driven by a particularly high number of unstained or sparsely stained airways in lung samples from patients with a UIP pattern. Most of these pauci-SCGB1A1 small airways displayed features of bronchiolar metaplasia. Interestingly, they were referred to as “bronchiolizing areas” in the routine pathology report.

Fukumoto et al. hypothesized that this phenomenon may indicate an impaired differentiation with aberrant proliferation (19). The latter hypothesis is supported by the morphologic appearance of these areas where proximal features are found where they should not be.

The underexpression of the club cell-specific protein SCGB1A1 may be directly linked to the bronchiolization process, as already supposed by Jensen-Taubman (20). A blockade in the notch pathway might be involved in this ontogenically impaired process, as the notch is critically involved in basal to club cell transitioning (21). Wnt/β-catenin pathways were also shown to be involved.

This mechanism may help accelerate the paradigm shift that is already in motion, where instead of envisioning bronchiolization and bronchiectasis as a consequence of fibrosis, they are now understood to be an active part of the fibrotic process [as again recently suggested (4)].

Senescence may play a key role in the fibrotic process characteristic of UIP (22) possibly *via* this bronchiolization. Aberrantly proliferating cells can lead to both replicative and premature senescence (23), possibly leading to these poorly differentiated airways. The secretion of senescence-associated secretory protein (SASP), mainly interleukin-8, has already been shown to be involved (24).

Our study has several limits. Few patients were included due to the particular procedures used in the CryoPID study. Our patient group was so heterogeneous that artificially gathering patients from a “non-UIP” group was required. Moreover, staining for MUC5B, a validated bronchiolization marker, would have been of great interest to increase confidence in the mechanism and further characterize the phenotype of the remaining club cells. As shown by Best et al. (25), a higher proportion of SCGB1A1^+^/MUC5B^+^ cells is observed in IPF, highly expressing genes related to mucins and chemoattractant cytokines for immune cells. Though we missed this opportunity, we nonetheless had attempted the Bleu Alcian staining in a few slides when some unused material was available; the latter suggested convergent findings and merits further investigation.

Of note, we decided to focus our analysis at the sample level (as opposed to the patient level) to take into account the regional heterogeneity of the fibrotic process where zones of normal appearance are classically neighboring the involved tissue (6). This was also justified by the aim of comparing TBLC and SLB in this regard. Correlations were tested at the individual level by expressing SCGB1A1 as a mean and our findings remained consistent.

There are a few steps left to definitively demonstrate that bronchiolization precedes the fibrotic process. Recently, by matching micro-CT imaging with histology, Verleden et al. showed that small airway involvement is the most striking finding in the early IPF stages, suggesting that the disease starts at this level (4).

Our study also supports that the bronchiolization process is key in the course of the disease as we could find correlations with two different HRCT-derived parameters. Kurtosis refers to the tailedness of the distribution histogram of lung attenuation, and this value was suggested to be a surrogate of interstitial changes (25). A significant negative relationship between the expression of the club cell marker SCGB1A1 and the bronchiectasis score was also found; an intuitively-expected finding enhancing the concept that bronchiectasis found at HRCT is related to the bronchiolization process hallmarked by the absence of club cells. Convincingly, a significant correlation was also found with FVC, a physiological marker more than relevant in interstitial lung diseases.

In summary, we demonstrated that bronchiolization, a typical characteristic of ILD and of UIP in particular, is also hallmarked by the absence of club cells. This suggests that an impaired transition process affected epithelial cell fate.

Pauci-SCGB1A1 staining status merits further study as a potential marker of bronchiolization in ILD.

## Data Availability Statement

The original contributions presented in the study are included in the article/supplementary material, further inquiries can be directed to the corresponding author.

## Ethics Statement

The studies involving human participants were reviewed and approved by CPP Ile de France. The patients/participants provided their written informed consent to participate in this study.

## Author Contributions

AB elaborated the study, enrolled patients, performed biopsies, participated to the interpretation of the data, and the writing of the manuscript. PR participated to the study, enrolled patients, performed biopsies, acquisition of data, immunostaining, morphometry, HRCT analysis, and the writing of the manuscript. EA participated to the study, enrolled patients, performed biopsies, acquisition of data, and the writing of the manuscript. IS participated to the study, assessed biopsies, and contributed to the immunostaining. LK, CV, CS, and IV participated to the study, interpretation of the data, and the writing of the manuscript. SB participated to HRCT analysis. MR, JB, JM, and AG participated to the study, enrolled patients, and performed biopsies. All authors contributed to the article and approved the submitted version.

## Conflict of Interest

The authors declare that the research was conducted in the absence of any commercial or financial relationships that could be construed as a potential conflict of interest.
